# Hepatitis C Virus Infection in Phenotypically Distinct Huh7 Cell Lines

**DOI:** 10.1371/journal.pone.0006561

**Published:** 2009-08-10

**Authors:** Bruno Sainz, Naina Barretto, Susan L. Uprichard

**Affiliations:** 1 Department of Medicine, University of Illinois at Chicago, Chicago, Illinois, United States of America; 2 Department of Microbiology and Immunology, University of Illinois at Chicago, Chicago, Illinois, United States of America; Yale University, United States of America

## Abstract

In 2005, the first robust hepatitis C virus (HCV) infectious cell culture system was developed based on the HCV genotype 2a JFH-1 molecular clone and the human-derived hepatoma cell line Huh7. Although much effort has been made to dissect and expand the repertoire of JFH-1-derived clones, less attention has been given to the host cell despite the intriguing facts that thus far only Huh7 cells have been found to be highly permissive for HCV infection and furthermore only a limited number of Huh7 cell lines/stocks appear to be fully permissive. As such, we compiled a panel of Huh7 lines from disparate sources and evaluated their permissiveness for HCV infection. We found that although Huh7 lines from different laboratories do vary in morphology and cell growth, the majority (8 out of 9) were highly permissive for infection, as demonstrated by robust HCV RNA and de novo infectious virion production following infection. While HCV RNA levels achieved in the 8 permissive cell lines were relatively equivalent, three Huh7 lines demonstrated higher infectious virion production suggesting these cell lines more efficiently support post-replication event(s) in the viral life cycle. Consistent with previous studies, the single Huh7 line found to be relatively resistant to infection demonstrated a block in HCV entry. These studies not only suggest that the majority of Huh7 cell lines in different laboratories are in fact highly permissive for HCV infection, but also identify phenotypically distinct Huh7 lines, which may facilitate studies investigating the cellular determinants of HCV infection.

## Introduction

Worldwide, ∼170 million individuals are infected with hepatitis C virus (HCV), an enveloped positive-strand RNA virus that infects and replicates in the hepatocytes of the liver. Although acute infection is typically asymptomatic, ∼80% of patients fail to clear the virus resulting in a chronic infection associated with significant liver disease, including cirrhosis and hepatocellular carcinoma [1].

Since its discovery in 1989 [2], the viral life cycle and host-virus interactions that determine infection outcome have been difficult to study because experimental HCV cell culture infection systems have been limited. Fortunately, in 2003 Wakita and colleagues cloned a HCV consensus genotype 2a genome (JFH-1) from a Japanese Fulminant Hepatitis patient [3], which was subsequently found to produce infectious HCV in Huh7 cell cultures [4]–[6]. This breakthrough was the result of two culminating factors: (1) identification of a HCV clone capable of replicating and assembling infectious particles in cell culture and (2) discovery that Huh7 cells are permissive for HCV infection. Surprisingly, however, significant variability in HCV permissiveness was observed among the original 3 Huh7 cell lines used to report HCV infection *in vitro* leading some to conclude that permissiveness for HCV may be limited to only a few Huh7 lines [7]. To test this hypothesis, we assembled a panel of Huh7 cell lines from disparate sources and assessed their permissiveness for HCV infection.

Here we show that although different Huh7 cell lines do vary in morphology and growth, 8 of 9 tested were highly permissive for HCV infection as demonstrated by robust HCV RNA and infectious virion production. Thus, this study illustrates that contrary to commonly held perception, HCV permissiveness does not appear to be restricted to only a few Huh7 cell lines. Furthermore, this effort has identified a panel of phenotypically distinct HCV-permissive Huh7 lines, which should facilitate studies investigating the cellular determinants of HCV infection.

## Materials and Methods

### Cells and viruses

Huh7-1 cells (also known as Huh7/scr cells [8], [9]) and Huh7.5.1 cells were obtained from Dr. Chisari (The Scripps Research Institute, La Jolla, CA) [6]. Huh7 cells designated Huh7-RRB were purchased from the Japan Health Science Research Resources Bank (JHSRRB, cat#JCRB0403). All other Huh7 cells were donated without restrictions by researchers outside the HCV research field, were blindly catalogued, and named Huh7-2 through Huh7-9; with the exclusion of Huh7-5 to avoid confusion with existing Huh7.5 [6], [10] and Huh7.5.1 cells [6]. These arbitrarily named and subsequently characterized cell lines are available upon request.

All cell lines were cultured in complete Dulbecco's modified Eagle's medium (DMEM) (Hyclone, Logan, UT) supplemented with 10% fetal bovine serum (FBS) (Hyclone), 100 units/ml penicillin, 100 mg/ml streptomycin, and 2 mM L-glutamine (Gibco Invitrogen, Carlsbad, CA). Cells were initially cultured for two consecutive weeks with Plasmocin (Invivogen, San Diego, CA) at 25 µg/ml, as per the manufacturer's instructions, to cure all cell lines of possible mycoplasma contamination. Confirmation of mycoplasma free cultures following treatment and throughout the course of this study was determined using the MycoProbe™ Mycoplasma Detection Kit (R&D Systems, Minneapolis, MN), as per the manufacturer's instructions (data not shown).

The genotype 2a JFH-1 plasmid (pJFH-1) has been previously described [5] and was kindly provided by Dr. Wakita (National Institute of Infectious Diseases, Tokyo, Japan). JFH-1 cell culture-propagated HCV (HCVcc) viral stocks were obtained by infection of naïve Huh7-1 cells at a multiplicity of infection (MOI) of 0.01 focus forming units (FFU)/cell, using medium collected from Huh7-1 cells on day 18 post transfection with *in vitro* transcribed pJFH-1 RNA as previously described [6]. Although HCV similarly collected at day 18 post transfection has been sequenced and determined not to contain any detectable adaptive mutations [9], we note that as with any propagated RNA virus, it is possible viruses containing adaptive mutations may be present within the HCV stock utilized.

### Growth kinetics

To ensure that none of the Huh7 cell lines would reach confluence during the 3 day period in which growth was monitored, a confluence of ∼40% was targeted by seeding ∼5×10^4^ Huh7 cells in each well of a 12-well tissue culture plate (Corning, Lowell, MA). At indicated times post seeding, medium from triplicate wells was removed, cells were washed once with 0.5 ml of 1X phosphate buffered saline (PBS), and trypsinized for 4 minutes at room temperature. Cells were resuspended to a final volume of 1 ml with complete DMEM and total cell numbers were determined using a hemacytometer.

### Viral propagation in different Huh7 cell lines

For each Huh7 cell line, 1.5×10^5^ cells were seeded in one T75 flask. Twenty four hours post seeding, medium was removed, cells were rinsed once with 10 ml of 1X PBS and then inoculated with JFH-1 HCVcc at an MOI of 0.01 FFU/cell in a total volume of 10 ml complete DMEM. Upon reaching confluence, cells were split 1∶3 until day 10 post-infection (p.i.). At indicated time points, supernatant was collected and infectivity titers determined by titration on naïve Huh7 cells.

### Infectivity titration assay

Culture supernatants were serially diluted 10-fold and used to infect duplicate 96-well Huh7 cultures. At 24 h p.i., cultures were overlaid with complete DMEM containing a final concentration of 0.25% methylcellulose (Fluka BioChemika, Switzerland). Seventy-two hours p.i., cells were fixed in 4% paraformaldehyde (Sigma, St. Louis, MO), and immunohistochemically stained for HCV E2 using the human monoclonal anti-E2 antibody C1 [11] (a gift from Dennis Burton, The Scripps Research Institute). Viral titers are expressed as FFU/ml, determined by the average E2-positive foci number detected at the highest HCV-positive dilution.

### HCV infection kinetics

Parallel cultures were infected with JFH-1 HCVcc at an MOI of 0.01 FFU/cell. Infected cells were trypsinzed before reaching confluence and re-plated at a 1∶3 dilution to maintain active growth. At indicated times p.i., medium was harvested from triplicate wells for titration and cellular RNA was isolated for reverse transcription followed by real-time quantitative PCR analysis (RTqPCR).

### RNA isolation and RTqPCR

Cellular RNA was isolated using an ABI PRISM™6100 Nucleic Acid PrepStation (Applied Biosystems). One µg of RNA was used for cDNA synthesis using TaqMan reverse transcription reagents (Applied Biosystems), followed by SYBRgreen RTqPCR using an Applied Biosystems 7300 real-time thermocycler as previously described [12]. HCV and GAPDH transcript levels were determined relative to standard curves of serially diluted JFH-1 or human GAPDH plasmids, respectively. Primers used are listed in Table S1.

### Pseudotyped retrovirus production and infections

Pseudotyped viruses were produced as previously described [13]. Briefly, DNA encoding HCV or vesicular stomatitis virus (VSV) glycoproteins were co-transfected with the Env-deficient HIV vector carrying a luciferase reporter gene (pNL4-3-Luc-R^-^-E^-^) into 293T producer cells. Supernatants were collected 48 h post transfection, filtered through a 0.45 µm-pore-size filter (BD Biosciences), aliquoted, frozen and subsequently tittered using the QuickTiter Lentivirus Titer Kit (Cell Biolabs, Inc., San Diego, CA) according to the manufacturer's instructions. Huh7 cells were inoculated with equal amounts of pseudotyped virus for 6 h, washed twice with 1X PBS, and at 72 h p.i. lysed in 20 µl of lysis reagent to measure luciferase activity (Promega, Madison, WI) using a FLUOstar Optima microplate reader (BMG Labtechnologies Inc, Durham, NC). For pseudotyped virus infections of transiently transfected cells, Huh7 cells were first electroporated (Biorad, Hercules, CA), as described [14], with plasmids expressing human CD81, scavenger receptor class B member 1 (SR-B1), claudin-1 (CLDN1) and/or occludin (10 µg total plasmid per transfection) 48 h prior to infection.

### Cellular dsRNA-response assay

Huh7 cells were mock-treated, treated with 100 U/ml IFN-β (PBL, New Brunswick, NJ), mock-transfected or transfected with 0.3 µg poly(I/C) (Sigma) or 100 ng pEGFP-N1 (Clontech, Mountain View, CA) using Lipofectamine™2000 (Invitrogen). Photographs were taken (Nikon Eclipse TE2000) 16 h post-transfection and cellular RNA was isolated for RTqPCR analysis of EGFP and IFN-stimulated genes (ISGs) (Primers listed in Table S1). EGFP transcript levels were normalized to GAPDH and copy numbers determined relative to a standard curve of serial dilutions of pEGFP-N1. Relative expression of ISGs was determined using the 2^−ΔΔ^C_T_ method [15], by normalizing to GAPDH expression levels.

## Results

### Morphological examination and growth kinetics

Since being isolated in 1982 from a Japanese male with a well-differentiated hepatocellular carcinoma [16], Huh7 cells have become widely used for the study of liver-related diseases and were fundamental to the development of the HCV infectious cell culture system [4]–[6]. Although commonly available, initial data suggested that Huh7 cells in distinct laboratories may differ substantially with respect to their ability to support HCVcc infection with only a minority supporting robust HCV infection [5], [7]. To test this hypothesis, we evaluated eight Huh7 lines obtained from disparate sources including the Huh7/scr cells (referred to here as Huh7-1), as these cells have been shown to be highly permissive for HCVcc infection [8], [9].

**Figure 1:**
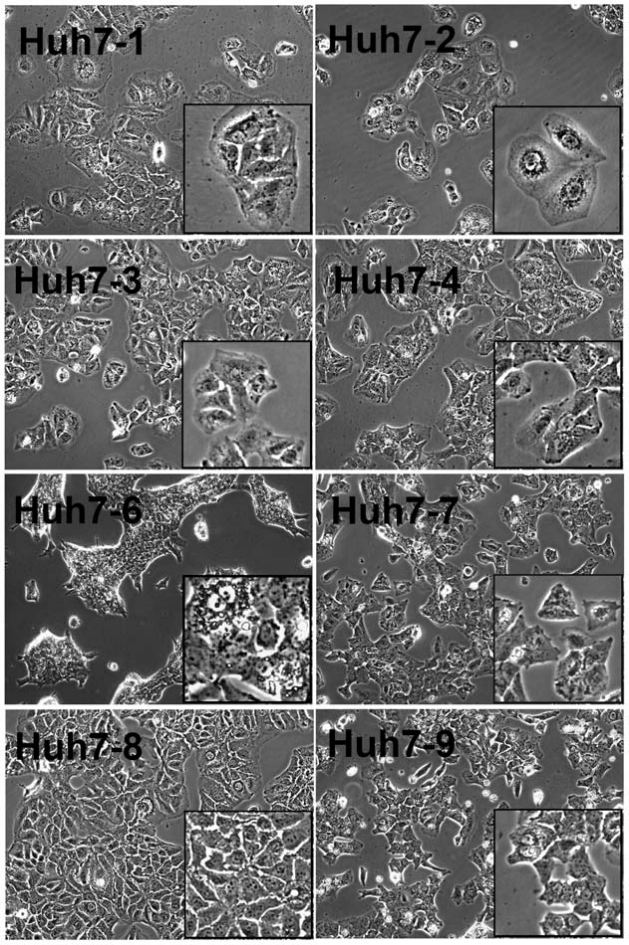
Morphological analysis of Huh7 cell lines. Huh7 cells were plated at 5×10^4^ cells/well in a 12-well plate and photographed 2 days after plating (magnification,×100; inset×400).

**Figure 2:**
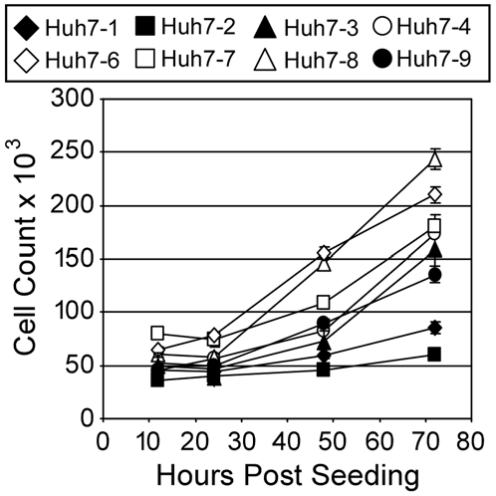
Growth kinetics of Huh7 cell lines. Cells were seeded at 5×10^4^ cells/well in a 12-well plate. At indicated times post-seeding, cells were trypsinized and counted. Results are graphed as mean±sem of triplicates.

Following a two month acclimation period of culturing under identical conditions, we evaluated the morphology (Figure 1) and growth kinetics (Figure 2) of each Huh7 line. Typical of hepatocytes, all cells grew in islands, exhibited cuboidal epithelial-like morphology with low nucleus-to-cytoplasm ratio, were mono- and bi-nucleated, and contained multiple nucleoli and cytoplasmic granules (Figure 1, insets). However, compared to one another, differences were evident. The Huh7-6 cells grew more tightly packed and formed multilayered islands morphologically distinct from the other cell lines which formed monolayer islands. Although all eight cell lines were hexagonal, the Huh7-1 and Huh7-2 cells were generally larger and more flattened, while the Huh7-8 cells exhibited the most uniform cuboidal shape.

To assess the growth rate of each Huh7 line, equal number of cells were plated in 12-well plates at ∼40% confluence and cell numbers determined at indicated times (Figure 2). By 48 h post-seeding, a difference in cell numbers was noted with distinct growth rates evident by 72 h. Specifically, the Huh7-3, -4, -7 and -9 cells had similar growth rates, while the growth rates of Huh7-1 and Huh7-2 cells were slower and Huh7-6 and Huh7-8 cells were faster (Figure 2).

Notably, although Huh7 cells are hepatoma cells, they do retain some hepatocyte characteristics such as expression of certain hepatocyte-specific genes [17]. As such, we confirmed the hepatocyte nature of all the cell lines by evaluating the levels of three hepatocyte-specific transcripts (hepatocyte nuclear factor 4-α, transthyretin, and α-1-antitrypsin) by real-time RTqPCR [18]. When compared to the Huh7-1 cells, no discernible difference in gene expression was detected (data not shown) confirming all the cell lines do share similar Huh7-like characteristics despite their morphological and growth differences.

### Propagation of HCV

**Figure 3:**
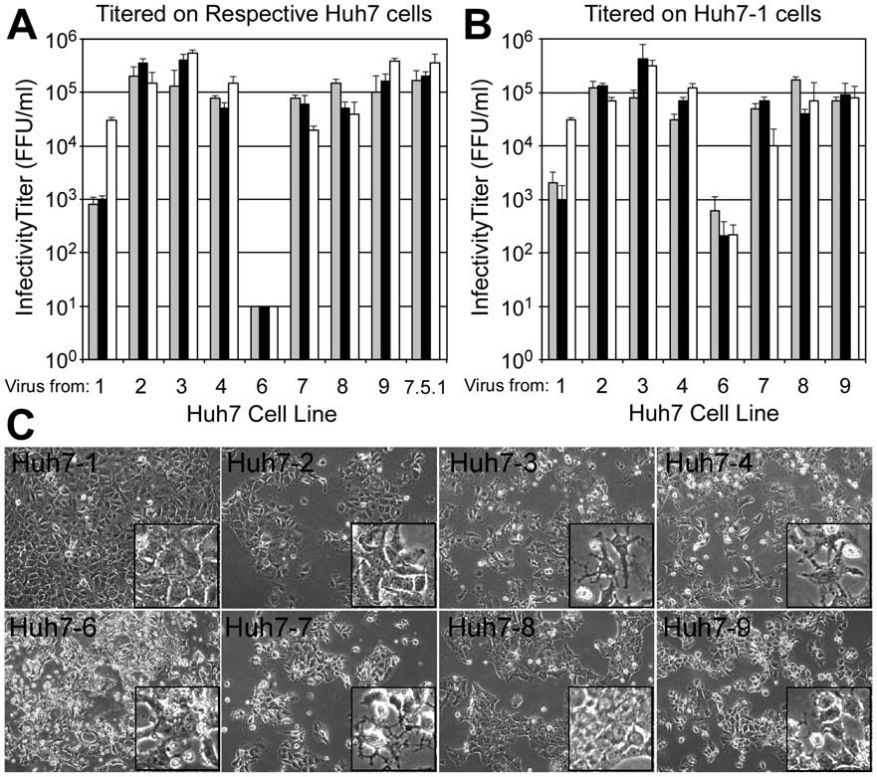
Propagation of infectious HCV. Cells were infected with JFH-1 HCVcc at an MOI of 0.01 FFU/cell and supernatant harvested on days 7 (grey bars), 8 (black bars) and 9 (white bars) p.i. Infectivity titers were determined on (A) the Huh7 cells from which the virus was derived and (B) Huh7-1 cells. Data represents three independent experiments. (C) HCV-induced CPE in Huh7 cells 8 days p.i. (magnification,×100; inset, ×400).

We next evaluated the ability of each cell line to propagate high titer HCVcc stocks. Based on previous studies, JFH-1 HCVcc titers were expected to peak 7-9 days p.i. [6]. As such, on days 7, 8 and 9 p.i., medium from HCVcc-infected cultures was collected and infectious virus levels titrated on the respective naïve Huh7 cells in which the virus was propagated. As expected, Huh7-1 cells efficiently propagated HCVcc with viral titers equal to 9×10^3^, 1.0×10^4^ and 5.0×10^4^ FFU/ml on days 7, 8 and 9 p.i., respectively while the Huh7.5.1 cells, which have been shown to propagate HCVcc to higher titers [6], produced HCVcc titers of 2.0×10^5^, 2.5×10^5^ and 5.0×10^5^ FFU/ml on days 7, 8 and 9 p.i., respectively (Figure 3A). Interestingly however, with the exception of the Huh7-6 cells, the other Huh7 cells similarly produced HCVcc to high titers, with titers greater than 5×10^4^ FFU/ml detectable as early as day 7 p.i. and titers ≥1×10^5^ FFU/ml by day 9 p.i. Notably, the Huh7-2, -3 and -9 cells supported virus production similar to that of Huh7.5.1 cells, with maximal titers of 1.5×10^5^, 6.1×10^5^ and 4.2×10^5^ FFU/ml detectable on day 9 p.i., respectively.

To eliminate any differences that might be a consequence of particular cell lines being more amendable to the focus-forming assay employed, we additionally titered all supernatants on the more routinely used Huh7-1 line [6]. However, no significant difference between titer values was observed on the Huh7 line in which the virus was generated (Figure 3A) or naïve Huh7-1 cells (Figure 3B). The only exception was Huh7-6 cells as this cell line does not support HCV infection sufficiently to be used for accurate titration analysis.

### HCV-mediated CPE

Although the degree and time of onset varied, all Huh7 lines exhibited some cytopathic effect (CPE) during infection, which included cessation of cell growth and accumulation of rounded and floating cells, similar to what has been reported in other Huh7 cells [9] (Figure 3C and Table 1). While minor CPE was observed in the Huh7-1 (and the relatively non-permissive Huh7-6 cultures), more profound CPE was apparent in the other six Huh7 lines (Figure 3C, insets), with the most rapid CPE occurring in Huh7-3, -7 and -9 cultures as early as day 5 p.i. leading to impairment of cell growth.

**Table 1:** Summary of Huh7 Cell Line Characteristics^a^.

Cell Line	CPE^b^	Average HCV Copies/µg RNA^d^	Average HCV Titers (FFU/ml)^e^	dsRNA Signaling^f^	JFH-1 Infectivity^g^
Huh7-1	+ (8)^c^	3.04×10^7^	4.67×10^4^	++++	++++
Huh7-2	++ (7)	2.68×10^7^	1.23×10^5^	+++	++++
Huh7-3	++++ (5)	2.94×10^7^	1.37×10^5^	++++	++++
Huh7-4	+++ (6)	2.53×10^7^	2.00×10^4^	++++	++++
Huh7-6	+ (8)	2.82×10^3^	3.67×10^1^	+++	+
Huh7-7	++++ (5)	2.35×10^7^	3.33×10^4^	++++	++++
Huh7-8	++ (7)	1.39×10^7^	1.00×10^4^	+++	++++
Huh7-9	++++ (5)	4.08×10^7^	1.43×10^5^	++	++++
Huh7-RRB	+ (8)	1.68×10^7^	7.11×10^4^	n/d	++++
Huh7.5.1	++++ (5)	2.44×10^7^	1.87×10^5^	n/d	++++

a Refer to Materials and Methods for definition of assays.

b CPE determined by microscopic examination. Changes in cell morphology, accumulation of floating cells and cessation of cell growth were scored based on the percentage of cells in the infected culture exhibiting these characteristics: +++, high (>80%); ++, medium (40–80%); + low (<40%); and undetectable.

c Day p.i. CPE was first evident.

d Average intracellular HCV RNA (copies/µg RNA) for days 6–8 p.i. (Figure 5A).

e Average infectivity titers (FFU/ml) for days 6–8 p.i. using respective Huh-7 cell line for assay (Figure 5B).

f Average ISG expression following poly(IC) transfection: ++++, >10-fold; +++, 3–10-fold; ++, <3-fold. (Figure 7B).

g HCV spread determined by indirect E2 immunofluorescence staining on day 7 p.i.: ++++, >90%; +++, 60–89; ++, 30–59%; +, <30% E2-positive cells (Figure S1).

n/d = not determined.

### Morphology of HCV foci

**Figure 4:**
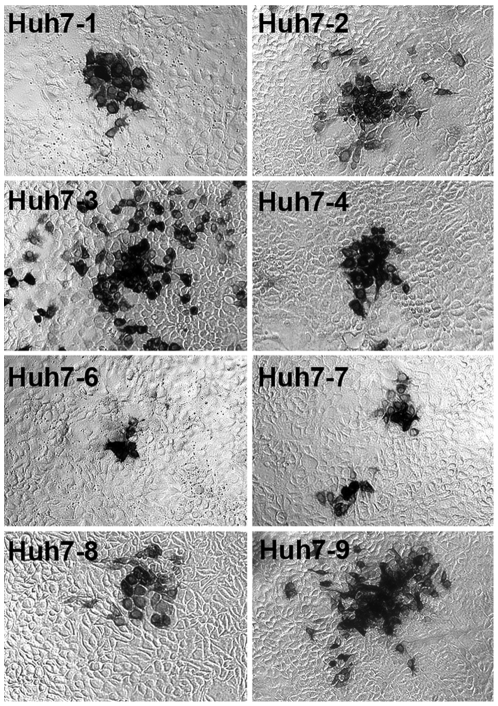
Phenotype of HCV-positive foci in different Huh7 lines during virus titration. Cells were overlaid with compete DMEM containing methylcellulose to reduce secondary spread. At 72 h p.i. cells were fixed and stained for HCV E2 [11] (magnification, ×100).

Interestingly, when titering infectious supernatants from the above experiment (Figure 3A) on the various Huh7 lines, we consistently noted that the phenotype of the foci formed on each cell line varied (Figure 4). The foci formed on Huh7-2, -3 and -9 cells were consistently larger, while the foci formed on Huh7-4 and -8 cells were equivalent in size to that observed in the Huh7-1 cultures, and the foci formed on Huh7-6 and -7 cells were noticeably smaller. Importantly, the same foci phenotypes were seen in the different Huh7 cells when a single HCVcc JFH-1 virus stock, propagated on Huh7-1 cells, was titered on all 8 Huh7 cell lines (data not shown). Likewise, no difference in foci size was observed when parallel Huh7-1 monolayers were inoculated with virus derived from the different Huh7 cells (data not shown). Hence, the differences in foci morphology were related entirely to the cell line used for titering and not to differences in the infecting virus stocks.

### Kinetics of HCV infection

**Figure 5:**
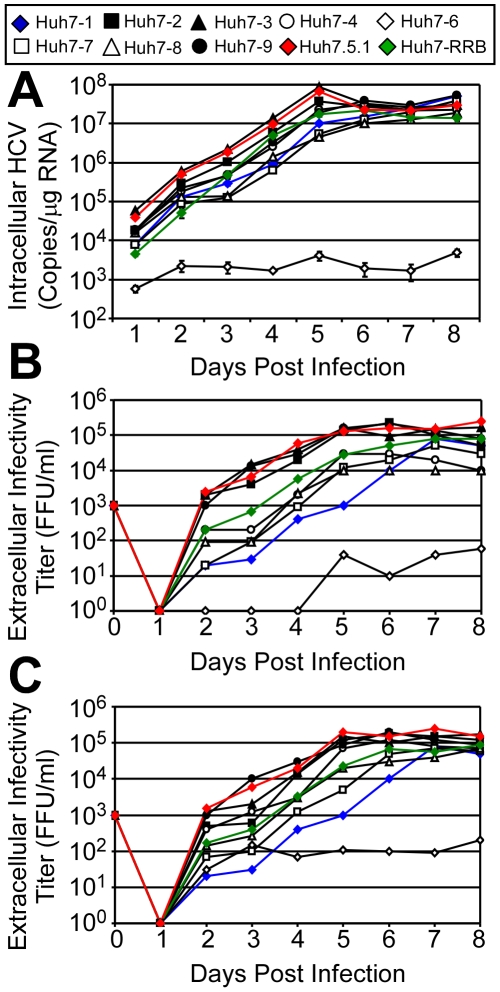
HCVcc replication kinetics. Cells were infected with JFH-1 HCVcc at an MOI of 0.01 FFU/cell. Culture supernatant and intracellular RNA were collected at the indicated times p.i. (A) Intracellular HCV RNA was analyzed by RTqPCR and is displayed as HCV copies/µg total RNA. Results are graphed as mean±sem of triplicates. Infectivity titers, expressed as FFU/ml, were determined on (B) the Huh7 cells from which the virus was derived and (C) Huh7-1 cells.

The difference in viral titers and size of HCV-positive foci observed among the various Huh7 cell lines suggested that some aspect of infection might be more efficient in certain Huh7 lines. Hence, we performed a kinetic analysis of HCVcc infection. Specifically, intracellular HCV RNA accumulation and release of infectious virus was examined daily for 8 days (Figure 5) in all the Huh7 lines as well as in Huh7.5.1 cells [6] and the original Huh7 cell line established by Nakabayshi et al. in 1982 [16], which were purchased from the JHSRRB (designated here Huh7-RRB) and cultured under similar conditions.

One day p.i., intracellular HCV RNA levels were similar among the majority of Huh7 lines (1.67×10^4^±6.10×10^3^ copies/µg RNA) exponentially increasing thereafter ∼1,500-fold by day 6 p.i., reaching a steady-state of 2.11×10^7^±4.44×10^6^ copies/µg RNA (Figure 5A and Table 1). Notably, HCV RNA expansion and plateau level reached was comparable among most Huh7 lines; however, HCV RNA copies in various cultures did vary up to one log on any given day with the Huh7-3 cells trending toward ∼5-fold greater levels days 2–6 p.i. The only exception was the lower level of HCV RNA produced in the Huh7-6 cells.

Virus production kinetics were determined by titering supernatants on both the Huh7 cell line from which the supernatant was harvested (Figure 5B) and Huh7-1 cells (Figure 5C). Unlike HCV RNA expansion, a difference in the initial levels and subsequent rate of virus production among the Huh7 cells was observed. Excluding Huh7-6 cells, Huh7-1 cells produced the least infectious virus with the slowest rate reaching maximal titers of 8×10^4^ FFU/ml by day 7 p.i. (Figure 5B–C). Although virus production rate from the Huh7-4, -7 -8, and -RRB cells closely paralleled that of the Huh7-1 cells, titers were consistently higher days 3-5 p.i. in these lines (Figure 5B). On the other hand, Huh7-2, -3 and -9 cells produced virus at significantly faster rates and higher levels, similar to that achieved in Huh7.5.1 cells (Figure 5B–C) and consistent with the propagation experiment above (Figure 3A–B and Figure 4). Notably, by day 2 p.i., titers of ∼10^3^ FFU/ml were detectable in the supernatant of these three cultures, 2 logs higher than Huh7-1 cultures, and subsequently reached maximal titers of ∼10^5^ FFU/ml by day 5 p.i.; two days before the other Huh7 lines (Figure 5B). Similar results were obtained when supernatants were titered on Huh7-1 cells (Figure 5C).

To more directly monitor cell-to-cell viral spread in the Huh7 cell lines, we also monitored HCV-positive cells at different times p.i. by immunofluorescence (Figure S1 and Supplementary Materials and Methods S1). Immunofluorescence staining for the HCV E2 glycoprotein indicated that in general, the percentage of E2-positive cells among the infected Huh7 cultures increased from ∼2% on day 2 to almost 100% by day 7 (Figure S1); however, the rate of spread (on days 4 to 7 p.i.) differed among the individual Huh7 cell lines, with the Huh7-2, -3 and -9 cells exhibiting accelerated spread kinetics. The percentage of E2-positive Huh7-2, -3 and -9 cells by day 5 p.i. was ∼90%, 95% and 85%, respectively, while in parallel less then 30% of the Huh7-1, -4, -7 and -8 cells and less than 2% of the Huh7-6 cells were E2 positive. This accelerated spread likely explains the faster rate of infectious virus production observed from these three cell lines (Figure 5B–C). Overall these results show that not only were the majority of these randomly acquired Huh7 lines highly permissive for HCVcc, but that permissiveness is a phenotype derived from the original Huh7 cells maintained by the JHSRRB, as the Huh7-RRB cells were equally permissive for HCVcc. Additionally, the data also indicate that the HCVcc-permissive Huh7 lines within this panel support varying levels of infectious progeny virus production.

### Huh7-6 cells are entry deficient

**Figure 6:**
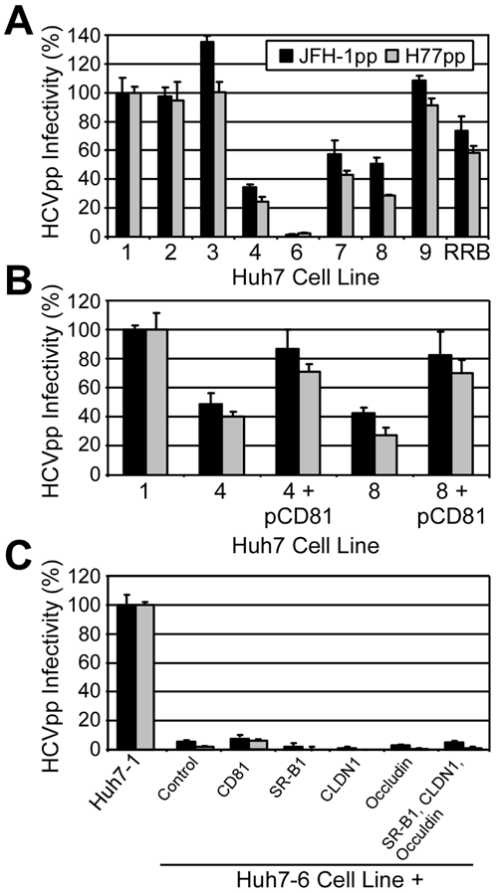
Huh7-6 cells are refractory for HCVpp infection. (A) Huh7 cells lines, (B) Huh7-4 and Huh7-8 cells transiently transfected with pEE6-huCD81, and (C) Huh7-6 cells transiently transfected with expression plasmids expressing CD81, SR-B1, CLDN1, and/or occludin, were infected with equal amounts of JFHpp, H77pp or VSVGpp. HCVpp infectivity titers (relative light units (RLU)±sem for triplicate samples determined 72 h p.i.), following background and mock-control signal subtraction, were divided by the titer of VSVGpp and compared to the signal in Huh7-1 cells.

To further characterize the Huh7 lines, including the refractory phenotype of the Huh7-6 cells, we tested the ability of the cells to support HCV pseudoparticle (HCVpp) entry and subgenomic JFH-1 (sgJFH-1) replicon replication, as these assays independently assess viral entry and replication, respectively. Hence, cells were infected with pseudotyped lentiviruses bearing HCV E1 and E2 of genotypes 2a or 1a (JFHpp and H77pp, respectively) or VSV G glycoprotein (VSVGpp). All cell lines were highly and equally permissive for VSVGpp infection; however, Huh7-4, -7 and -8 cells showed a 50–60% reduction in HCVpp entry, while Huh7-6 cells were 90% less permissive as compared to Huh7-1 cells (Figure 6A); suggesting HCV entry is significantly impaired in Huh7-6 cells. In contrast, when entry was bypassed, we detected no difference in sgJFH-1 colony formation efficiency among the different Huh7 lines (Figure S2 and Supplementary Materials and Methods). Taken together, these observations indicate that a defect in HCV entry likely explains the relatively non-permissive nature of the Huh7-6 cells. Interestingly, reduced levels of HCV entry in the Huh7-4, -7 and Huh7-8 cells may also explain the lower level of early virus production (Figure 5B) observed with these three HCVcc-permissive Huh7 cell lines.

### Cell surface expression of HCV receptors

To functionally assess if reduced surface expression of one of the putative cellular HCV receptors might be responsible for the low HCVcc and HCVpp infectivity of the Huh7-6 cells, flow cytometric analysis of CD81, low density lipoprotein-receptor (LDL-R) and SR-B1 was performed (Figure S3 and Supplementary Materials and Methods). Interestingly, while most of the Huh7 cell lines expressed similar and uniformly high levels of CD81 on their cell surface, a bi-modal CD81 expression profile was observed for Huh7-4 and Huh7-8 cells (Figure S3A), similar to what was reported by Koutsoudakis et al. for the relatively non-permissive Huh7-Lunet cells [19]. Surface level expression of LDL-R and SR-B1 did not vary among the Huh7 cell lines except for the Huh7-6 cells, which had undetectable levels of SR-B1 (Figure S3B–C).

In light of these results, we transiently transfected Huh7-4 and -8 cells as well as Huh7-6 cells with vectors expressing human CD81 or SR-B1, respectively, and reassessed their permissiveness for HCVpp infection 48 h post-transfection. As shown in Figure 6B, expression of CD81 *in trans* significantly improved HCVpp permissiveness to the Huh7-4 and -8 cells; however, SR-B1 complementation did not rescue Huh7-6 cells (Figure 6C). Likewise, permissiveness to HCVpp could not be conferred to the Huh7-6 cell by expression of a panel of known HCV receptors, alone or in combination, suggesting that Huh7-6 cells may be defective in another currently unidentified HCV entry determinant (Figure 6C).

### Responsiveness of the dsRNA-signaling pathway in Huh7 cell lines

**Figure 7:**
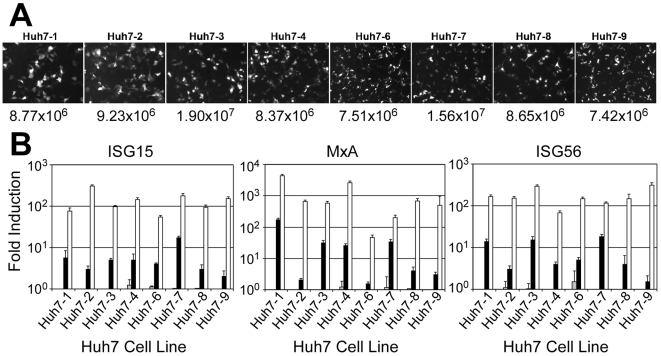
ISG induction in Huh7 lines. (A) EGFP expression in each Huh7 cell line 16 h post-transfection with 100 ng pEGFP-N1. EGFP protein visualized by Axiovert 200 fluorescence microscope. EGFP mRNA determined by RTqPCR (bottom). (B) In parallel, additional wells were mock-treated (grey bars), transfected with 300 ng poly(I/C) (black bars) or treated with 100 U/ml IFN-β (white bars). Cellular RNA was harvested 16 h post treatment and ISG expression was assessed by RTqPCR. Values are expressed as mean fold-induction±sem for triplicate samples compared to untreated controls.

Finally, because abrogation of the cellular RIG-I/interferon regulatory factor 3 (IRF-3) pathway can confer an advantage to HCVcc in vitro (e.g. the Huh-7.5 cells [20]), we evaluated the response of each Huh7 line to double-stranded RNA (dsRNA), an inducer of this pathway [21], [22]. Specifically, we transfected each Huh7 line with 300 ng poly(I:C) and evaluated representative IFN-inducible gene (ISG) transcript levels (ISG15, MxA and ISG56) by RTqPCR. As a positive control for ISG induction, separate cultures were treated with 100 U/ml IFN-β. To confirm equivalent transfection efficiency, cells were additionally transfected with 100 ng pEGFP-N1 and EGFP protein and mRNA was evaluated 16 h post-transfection. Importantly, no significant differences in EGFP protein or mRNA levels were observed among the Huh7 cell lines following transfection (Figure 7A) and all eight lines responded to IFN-β treatment (Figure 7B).

The level of poly(I:C)-induced ISG expression, however, did vary among the cell lines. Specifically, poly(I:C)-induced ISG up-regulation was ≥10-fold in the Huh7-1, -3 and -7 cells, while the Huh7-2, -6, and -9 cells exhibited ≤5-fold induction compared to mock-transfected controls (Figure 7B). Thus, it is possible that lower dsRNA responsiveness may in part explain the enhanced HCVcc production in Huh7-2 and Huh7-9 cultures. Notably however, the higher dsRNA-induced ISG expression observed in the Huh7-3 cells did not seem to limit its capacity to support HCV infection leading us to conclude that dsRNA-responsiveness must not be the universal factor responsible for the differences in infectious HCVcc production observed among these Huh7 cell lines, as suggested for the Huh7.5 cells [20].

## Discussion

In this study we characterized eight Huh7 cell lines at the level of growth, morphology, and HCVcc permissiveness. As summarized in Table 1, we show that in general all these Huh7 cells as well as the original Huh7 cells preserved at JHSRRB are inherently permissive for HCVcc infection; however, specific differences were evident.

### Phenotypic drift of continuous cell lines

Cell lines kept in culture can functionally evolve over time to no longer represent the original cell line (reviewed in [23]). Since their isolation in 1982 [16], this phenomena appears to have occurred with some Huh7 cell lines. For example, Wakita and colleagues found their Huh7 cell line to be relatively non-permissive [5] and determined this was because most of the cells were CD81-negative [24]. Notably, the one Huh7 cell line we found to be relatively non-permissive, the Huh7-6 cell line, has also down-regulated an essential HCV receptor, SR-B1, and perhaps another yet unidentified entry factor. As a whole, however, the majority of the Huh7 lines analyzed here maintained their inherent permissiveness to HCVcc infection although difference in growth kinetics and general morphology were apparent (Figure 1 and Figure 2).

### Permissiveness of Huh7 cell lines for HCVcc and HCVpp infection

Considering the phenotypic differences observed among the eight Huh7 cell lines collected for this study and based on previous reports demonstrating low HCVcc infection efficiency in various Huh7 cell lines [19], [24], we were surprised that 7 of the 8 Huh7 lines tested efficiently propagated HCVcc (Figure 3A–B). In fact, the level of de novo viral production for three of the cell lines (i.e. Huh7-2, -3 and -9) was equal to that achieved by the “adapted” cell line, Huh7.5.1 [6] (Figure 3A).

#### (i) Differences in HCVcc foci formation

HCV-positive foci size varied among the different Huh7 cell lines with larger foci apparent in the Huh7-2, -3 and -9 cell lines (Figure 4), all three of which propagated HCVcc to titers greater than that of the other Huh7 cell lines (Fig. 3). More rapid cell division during the 3 day assay does not appear to explain the increased foci size, since the Huh7-2 cells demonstrated one of the slowest proliferation rates. Hence, the size of the foci observed is more likely related to differences in the rate of de novo viral production and/or spread.

#### (ii) Comparable HCVcc RNA amplification

Despite the obvious difference in foci size, HCV RNA exponentially expanded with similar kinetics and eventually reached comparable plateau levels (∼2.3×10^7^ HCV copies/µg RNA) in the different infected Huh7 cell cultures, with the exception of the Huh7-6 cells which consistently supported lower levels of HCV infection (Figure 5A and Table 1). Similar RNA plateau levels were also reached in the Huh-7.5.1 cells (Figure 5A). One possible explanation for the observed plateau may be that Huh7 cells only have the capacity to support a given level (∼1.0×10^7^ copies/µg RNA) of HCV RNA replication and this phenotype is maintained among the different cell lines. Alternatively, the maximum RNA level obtained may be determined by inherent life cycle regulatory strategies encoded by the virus.

#### (iii) Differences in infectious HCVcc production

Although little variability was observed in HCV RNA levels in the different Huh7 cultures, a higher degree of divergence was observed in infectious virion production (Figure 5B–C). While, the Huh7-1, -4, -7, -8 and -RRB cells demonstrated comparable levels, the Huh7-2, -3 and -9 cells secreted infectious HCVcc sooner and to higher titers, similar to that observed in Huh7.5.1 cells (Figure 5B–C). Hence, it will be interesting to determine the mechanism(s) that mediate what appears to be more efficient de novo virus production in the Huh7-2, -3 and -9 cells (e.g. packaging, maturation and/or secretion) as such studies may identify critical cellular factors involved in these processes. Of note, viral entry-independent assessment of early post-entry events (e.g. translation and RNA replication) can be performed by transfecting *in vitro* transcribed infectious JFH-1 HCV RNA into Huh7 cells. However, this experimental strategy would be less conclusive when addressing later, post-replication events such as de novo HCVcc production because the HCVcc “transfection-dependent” titers achieved at early time points post-transfection are extremely low and only become reliably detectable 48-72 h post-transfection after rounds of “entry-dependent” viral spread have occurred.

#### (iv) Permissiveness for HCVpp infection

While only the Huh7-6 cell line showed reduced permissiveness for HCVcc, we identified four cell lines (Huh7-4, -6, -7 and -8) that exhibited a reduction in permissiveness for HCVpp. Although such a reduction could easily explain the relatively non-permissive phenotype of the Huh7-6 cells for HCVcc infection, none of the other three cell lines showed impaired HCVcc infection kinetics, particularly at early time points p.i. (Figure 5A and Figure S1). This observation would suggest that the requirements for entry may differ between the HCVpp and HCVcc systems.

### Identifying cellular determinants of HCV permissiveness

To determine if the differences in HCVpp and HCVcc infection could be attributed to factors already shown to modulate HCV permissiveness in other Huh7 cell lines [4], [6], [19], [20], [24]–[27], we examined the expression of HCV entry receptors (e.g. CD81, LDL-R or SR-B1) and IFN responsiveness to dsRNA. Interestingly, the data suggests that in this panel of Huh7 cells, CD81 expression levels more significantly influence HCVpp rather than HCVcc permissiveness. Specifically, the Huh7-4 and -8 cell lines exhibited a bimodal CD81 expression pattern (Figure S2A), which significantly reduced their permissivity for HCVpp (Figure 6A). This bimodal CD81 expression pattern is reminiscent of that observed for the Huh7-Lunet cells [19]; however, although bimodal CD81 levels restricted HCVcc infection in the Huh7-Lunet cells, Huh7-4 and -8 cells were highly permissive for HCVcc infection (Figure 5). The lack of reduced HCVcc infectivity associated with bi-modal CD81 expression observed here may be due to other differences between the cell lines or between the HCV clone used by Koutsoudakis et al. (Jc1) versus this study (JFH-1) [19]. As such, it would be interesting to determine the permissiveness of Huh7-4 and Huh7-8 cells for Jc1-HCVcc to ascertain if the critical surface density level of CD81 required for entry varies among HCV clones [19]. Nonetheless, there does appear to be a difference in dependency for CD81 between HCVcc and HCVpp, which highlights an inherent difference between these two model systems.

In terms of SR-B1 cell surface expression, only the Huh7-6 cell line had undetectable levels of this critical HCV entry factor (Figure S2C); however, complementation of Huh7-6 cells with exogenous SR-B1 expression alone did not restore its permissiveness for HCVpp (Figure 6C) or HCVcc (data not shown) infection, suggesting that although Huh7-6 cells lacked detectable cell surface expression of SR-B1, this receptor alone was not the limiting factor precluding HCVpp entry. Since claudin-1 [28] and occludin [29], [30] have also been shown to be necessary for HCVpp and HCVcc entry, and since ectopic expression of these receptors confers HCV permissiveness to non-permissive cells, we additionally tested the ability of these receptors, alone or in combination with SR-B1, to complement Huh7-6 cells, but none of the receptor combinations tested restored HCV infection to Huh7-6 cells (Figure 6C), suggesting that another yet unidentified HCV entry factor may exist. As such, future studies to dissect and evaluate why HCV entry into the Huh7-6 cells is limited seem warranted.

Since attenuation of the RIG-I/interferon regulatory factor 3 (IRF-3) pathway is believed to confer an advantage to HCVcc in Huh7.5 and Huh7.5-derived cells [4], [6], [20], we tested whether these eight Huh7 cell lines responded differently to dsRNA. Although all cells responded well to IFN-β stimulation, obvious differences in dsRNA responsiveness were apparent, with 3 out of the 8 cell lines showing reduced responsiveness to poly(IC) stimulation (Figure 7B), similar to that of Huh7.5.1 cells (data not shown). Of interest, lower induction of IFN-induced genes in response to dsRNA was observed in two of the most permissive cell lines, Huh7-2 and Huh7-9 suggesting that defects in dsRNA signaling may be contributing to the high HCV levels achieved in these cells. However, attenuated responsiveness to dsRNA does not necessarily limit HCVcc infection in specific cell lines because the Huh7-3 cell line, which supported equally robust HCVcc infection, showed high IFN stimulated gene induction in response to dsRNA. Such seeming discordances are in agreement with a recent study by Binder et al showing no correlation between HCV permissiveness and inducibility of the RIG-I/IRF-3 pathway in several Huh7 cell lines and cured replicon clones [21]. Taken together, it appears as though the absence of a functional RIG-I/interferon regulatory factor 3 (IRF-3) pathway may not be critical for HCV replication as previously hypothesized.

### Identifying cellular determinants of HCV-mediated cytopathic effect

HCV has long been regarded as a non-cytopathic virus; however, the observation that HCVcc can induce CPE in Huh7 cells suggests that at sufficient levels HCV can be deleterious to the cell [6]. During propagation of HCVcc, we noted varying degrees of CPE in the different Huh7 cell lines (Figure 3C), which did not simply correlate with the level of HCV replication. For example, the Huh7-2 cell propagated HCV to similar levels as the Huh7-3 cells, but only the Huh7-3 cells exhibited high HCV-induced CPE. With recent studies indicating that HCV can directly induce apoptosis of infected cells via a Bax-triggered, mitochondrion-mediated, caspase 3-dependent pathway and/or a TRAIL-induced caspase 9-dependent manner [31], [32], identifying differential activity of these and/or other cellular pathways in the different Huh7 cell lines may help better dissect the mechanisms responsible for HCV-mediated CPE.

In summary, these data provide us with a characterized panel of HCVcc permissive and non-permissive Huh7 cells. While this study confirms that changes in HCV entry factors represent a common reason for reduced permissiveness to HCVpp and HCVcc infection and that reduced responsiveness to dsRNA can be associated with higher HCVcc infection levels, these cell lines display differences in infectious HCVcc production independent of these previously identified cellular determinants and hence should prove useful for elucidating other critical Huh7 cell factors involved in HCV infection and HCV-mediated cytotoxicity. Importantly, the differences observed among these independently acquired Huh7 cell lines also highlights that in some cases, phenotypic differences between Huh7 cells may need to be considered when comparing HCV studies across laboratories. Although these differences raise questions regarding whether the commercially available original Huh7 cells (Huh7-RRB) should be adopted as some type of “standard” in the field, the comparison of HCVcc infection dynamics in multiple Huh7 cell lines also clearly can provide insight into the viral-host interactions required for infection.

## Supporting Information

Supplementary Materials and Methods S1 (0.07 MB PDF)

Table S1 Primer Sequences for RTqPCR
(0.02 MB PDF)

Figure S1 Kinetics of HCV spread and protein expression in Huh7 cell lines. Indirect immunofluorescence analysis of HCV E2 protein expression in Huh7 cells infected at an MOI of 0.01 FFU/cell and cultured for 7 days p.i. HCV E2 is red (Alexa 555) and nuclei are blue (Hoechst). Magnification×100.
(16.25 MB TIF)

Figure S2 Subgenomic HCV G418-resistant colony formation in Huh7 cell lines. One µg of in vitro transcribed sg JFH-1 RNA was electroporated into 2×10^6^ cells of each Huh7 cell line. Cells were diluted 1∶500 and plated in 6-well plates, in triplicate, and maintained in the presence of 500 µg/ml G418 for 2 weeks. Colonies were fixed, stained with crystal violet and photographed.
(1.06 MB PDF)

Figure S3 Flow cytometric analysis of HCV putative receptor surface expression on Huh7 cell lines. Each Huh7 cell line was stained with (A) mouse anti-CD81, (B) mouse anti-LDL-R or (C) rabbit anti-SR-B1 monoclonal antibodies and respective anti-mouse or anti-rabbit secondary antibodies conjugated with PE. Shaded regions represent cells stained with a monoclonal mouse or rabbit IgG control primary antibody and respective PE-conjugated secondary antibody.
(2.14 MB TIF)
