# Supplementary material for: Hepatitis C Virus Infection in Phenotypically Distinct Huh7 Cell Lines
Source: PLoS One. 2009 Aug 10;4(8):e6561. doi: 10.1371/journal.pone.0006561 (PMC2720605; doi:10.1371/journal.pone.0006561)
Supplement: Supplementary Materials and Methods S1 — (0.07 MB PDF) [file pone.0006561.s001.pdf]

Hepatitis C Virus Infection in Phenotypically Distinct Huh7 Cell Lines

Bruno Sainz, Jr. [1], Naina Barretto[1] and Susan L. Uprichard[1,2*]

[1]Department of Medicine, University of Illinois at Chicago, Chicago, IL, USA.

[2]Department of Microbiology and Immunology, University of Illinois at Chicago, Chicago, IL, USA.

Supplementary Materials and Methods

Indirect immunofluorescence. Eight-well chamber slides (Nunc, Roskilde, Denmark) were seeded with 9,000 cells/chamber of each Huh7 cell line. Twenty-four post seeding, cells were infected with JFH-1 HCVcc at an MOI of 0.01 FFU/cell in a total volume of 0.3 ml complete DMEM. Infected cells were trypsinized before reaching confluency and replated at a dilution of 1:3 when necessary days 3-6 p.i. At indicated days p.i., one chamber slide was fixed with 4% paraformaldehyde (Sigma) and intracellular staining was performed. Briefly, the human monoclonal anti-HCV E2 antibody C1 was used at a dilution of 1:500 followed by incubation with a 1:1,000 dilution of an Alexa555-conjugated goat anti-human IgG antibody (Molecular Probes, Carlsbad, CA) for 1 h at room temperature. Cell nuclei were stained by Hoechst dye. Chambers were removed, stained cells were coverslipped and mounted in Prolong Antifade mounting medium (Molecular Probes) and visualized on a Nikon Eclipse TE2000 microscope (Nikon Corp., Thornwood, NY) using Metamorph software (Molecular Devices). Images were generated with Adobe Photoshop (Adobe Systems Inc., San Jose, CA).

HCV RNA transfection and G418-resistant colony formation. HCV subgenomic (sg) 2a replicons were established as previously described [1]. Briefly, 1 µg of *in vitro* transcribed HCV genotype sg2a replicon RNA (psgJFH-1 was kindly provided by T. Wakita, National Institute of Infectious Diseases, Tokyo, Japan) was transfected into cells using a modified electroporation protocol [2]. Transfected cells were diluted 1:500, seeded in 6-well plates and maintained in the presence of G418 (Invitrogen) at a concentration of 500 µg/ml until all

cells died or distinct G418-resistant cell colonies formed. To visualize colony formation, cells were fixed and stained with crystal violet.

Flow cytometric analysis of cell surface CD81, LDL-R and SR-BI. One million cells of each Huh7 cell line were resuspended in 150 µl of FACS buffer (1X PBS containing 2% (v/v) FBS, 0.3% (w/v) NaN_3 and 1mM EDTA) and incubated for 60 min at 4°C with a 1:100 dilution of antibodies specific for CD81 (Serotec, Raleigh, NC), LDL-R (BD Pharmingen), or SR-BI (Novus Biologicals, Littleton, CO). Following three rinses with FACS buffer, bound antibodies were detected by incubation for 1 h at 4°C with phycoerythrin (PE)-conjugated anti-mouse (BD Pharmingen) (for CD81 and LDL-R) or anti-rabbit (Santa Cruz Biotechnology, Santa Cruz, CA) (for SR-B1) antibodies at a dilution of 1:200. Cells stained with irrelevant immunoglobulin G (IgG) antibodies and respective PE-conjugated secondary antibody served as negative controls. Cells were washed three times, fixed in FACS buffer containing 4% (w/v) PFA, and analyzed by flow cytometry using the DakoCytomation CyAn system (Dako, Carpinteria, CA) and Summit Software v4.3 (Dako).

DNA constructs. The human CD81 expression plasmid (pEE6-huCD81) has been previously described [3]. The human SR-BI expression plasmid (pZeo_hSR-B1) was cloned by PCR amplifying the human SR-BI ORF with primers 5'AGG CAA GCT TGC CGC CAT GGG CTG CTC CGC CAA AGC GCG CTG GG 3' (sense) and 5' CCA GTC TAG ACT ACA GTT TTG CTT CCT GCA GCA CAG AGC CC 3' (anti-sense). This product was cloned as a HindIII/XbaI fragment into HindIII/XbaI-digested pZeoSV2(-) (Invitrogen). The human CLDN1 expression plasmid (pZeo_CLDN1) was cloned by PCR amplifying the human CLDN1 ORF with primers 5' GCG GCC GCA TGG CCA ACG CGG G 3' (sense) and 5' GCG GCC GCT CAC ACG TAG TCT TTC CCG CTG G 3' (anti-sense). This product was cloned as a NotI fragment into NotI-digested pZeoSV2(-) (Invitrogen). The human occludin expression plasmid (pCDNA3.1_OCLN) was cloned by PCR amplifying the human occludin ORF with primers 5' GGG CTA GCA TGT CAT CCA GGC CTC TTG 3' (sense) and 5' ACC CTC GAG CTA TGT TTT CTG TCT ATC 3' (anti-sense). This product was cloned as a Xho/NheI fragment into Xho/NheI-digested pZeoSV2(-) (Invitrogen). The JFH-1 E1/E2 glycoprotein expression plasmid (pCDNA3.1_JFHcE1/E2) was cloned by PCR amplifying the polyprotein residues 167-751 of pJFH-1 with primers 5'-GAA TTC ATG GGG AAC CTA CCC GG-3' (sense) and 5'-CTC TAG ACT ATG CTT CGG CCT GG-3' (anti-sense).

This product was cloned as an EcoRI/XbaI fragment into EcoRI/XbaI-digested pCDNA3.1 (Invitrogen). The H77 E1/E2 glycoprotein expression plasmid (pCDNA3.1_H77cE1/E2) was cloned by PCR amplifying the polyprotein residues 170-746 of pH77 with primers 5'-GAA TTC ATG GAT GAT CTG GAC G -3' (sense) and 5'-CTC TAG ACT AGA CGT CGA AGC TG-3' (anti-sense). This product was cloned as an EcoRI/XbaI fragment into EcoRI/XbaI-digested pCDNA3.1 (Invitrogen). The VSV G glycoprotein expression plasmid (pCDNA3.1_VSVG) was cloned by PCR amplifying the VSVG coding sequence from pET-3c (Novagen, Gibbstown, NJ) with primers 5'- GAA TTC ATG AAG TGC CTT TTG TAC TTA GCC -3' (sense) and 5'-CTC TAG ATT ACT TTC CAA GTC GGT T-3' (anti-sense). This product was cloned as an EcoRI/XbaI fragment into EcoRI/XbaI-digested pCDNA3.1 (Invitrogen).

Supplementary References

1. Uprichard SL, Chung J, Chisari FV, Wakita T (2006) Replication of a hepatitis C virus replicon clone in mouse cells. Virol J 3: 89.
2. Krieger N, Lohmann V, Bartenschlager R (2001) Enhancement of hepatitis C virus RNA replication by cell culture-adaptive mutations. J Virol 75: 4614-4624.
3. Higginbottom A, Quinn ER, Kuo CC, Flint M, Wilson LH, et al. (2000) Identification of amino acid residues in CD81 critical for interaction with hepatitis C virus envelope glycoprotein E2. J Virol 74: 3642-3649.
