# Supplementary material for: Hepatitis C Virus Infection in Phenotypically Distinct Huh7 Cell Lines
Source: PLoS One. 2009 Aug 10;4(8):e6561. doi: 10.1371/journal.pone.0006561 (PMC2720605; doi:10.1371/journal.pone.0006561)
Supplement: Table S1 — Primer Sequences for RTqPCR (0.02 MB PDF) [file pone.0006561.s002.pdf]

Supplementary Table 1. Primer Sequences for RTqPCR

Gene	Accession No.	Forward Primer	Reverse Primer
JFH-1	AB047639	TCTGCGGAACCGGTGAGTA	TCAGGCAGTACCACAAGGC
GAPDH	NM_X002046	GAAGGTGAAGGTCGGAGTC	GAAGATGGTGATGGGATTTC
EGFP	U55762	GCAAAGACCCCAACGAGAAG	TCACGAACTCCAGCAGGACC
ISG15	NM_005101	CAGCGAACTCATCTTTGCCAGTA	CCAGCATCTTCACCGTCAGG
ISG56	NM_001001887	GGGCAGACTGGCAGAAGC	TATAGCGGAAGGGATTTGAAAGC
MxA	NM_002462	CCAGCATCTTCACCGTCAGG	CCCTTCTTCAGGTGGAACAC
Transthyretin (TTR)	NM_000371	CCGGTGAATCCAAGTGTCCT	GCACGGCCACATTGATG
HNF4α	Z49825	ACATTCGGGCGAAGAAGATT	ACTTGGCCCACTCAACGAG
α1-antitrypsin (A1AT)	M11465	TGCTGCCCAGAAGACAGATA	GGCGGTATAGGCTGAAGG
