# Supplementary figures and images for: Hepatitis C Virus Infection in Phenotypically Distinct Huh7 Cell Lines
Source: PLoS One. 2009 Aug 10;4(8):e6561. doi: 10.1371/journal.pone.0006561 (PMC2720605; doi:10.1371/journal.pone.0006561)

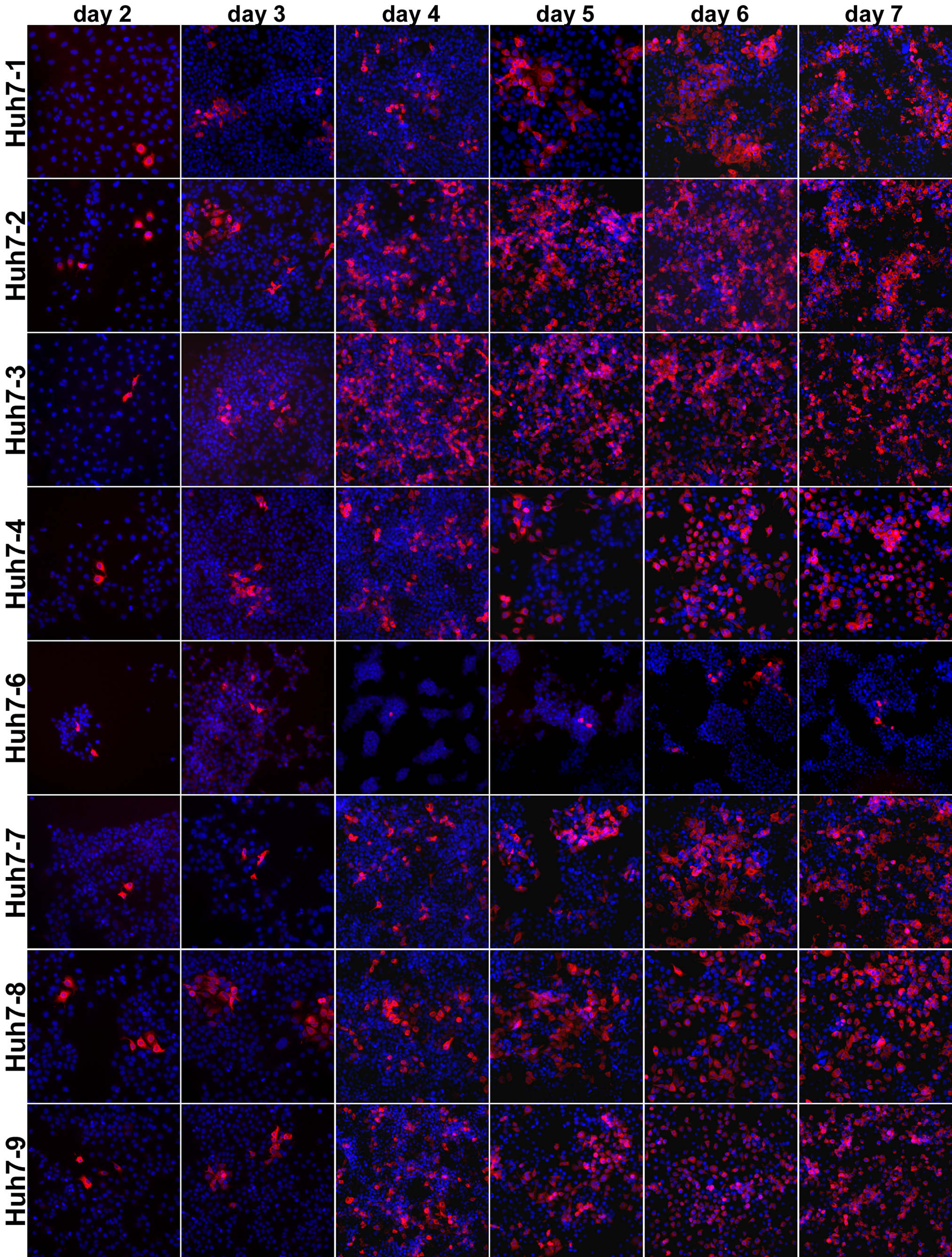

Supplement: Figure S1 — Kinetics of HCV spread and protein expression in Huh7 cell lines. Indirect immunofluorescence analysis of HCV E2 protein expression in Huh7 cells infected at an MOI of 0.01 FFU/cell and cultured for 7 days p.i. HCV E2 is red (Alexa 555) and nuclei are blue (Hoechst). Magnification×100. (16.25 MB TIF) [file pone.0006561.s003.tif]

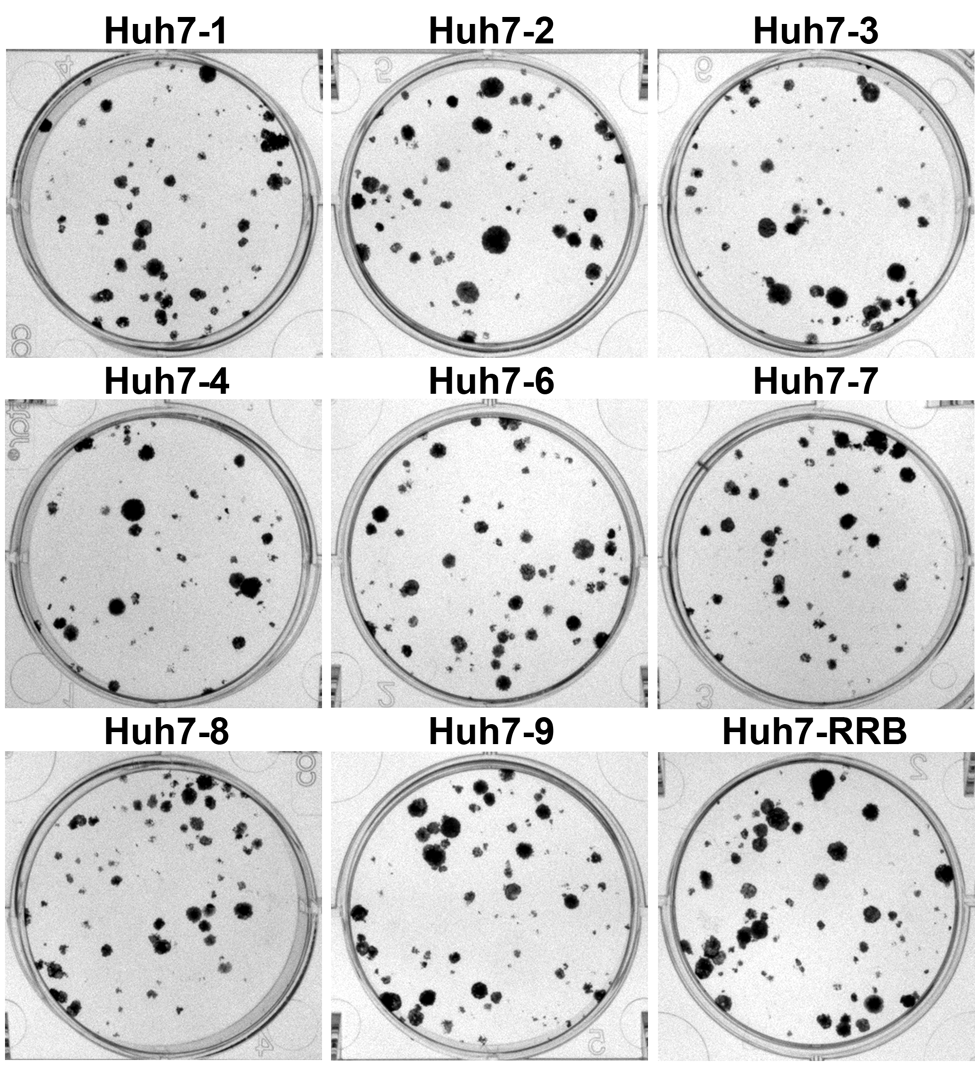

Supplement: Figure S2 — Subgenomic HCV G418-resistant colony formation in Huh7 cell lines. One µg of in vitro transcribed sg JFH-1 RNA was electroporated into 2×10^6^ cells of each Huh7 cell line. Cells were diluted 1∶500 and plated in 6-well plates, in triplicate, and maintained in the presence of 500 µg/ml G418 for 2 weeks. Colonies were fixed, stained with crystal violet and photographed. (1.06 MB PDF) [file pone.0006561.s004.tif]

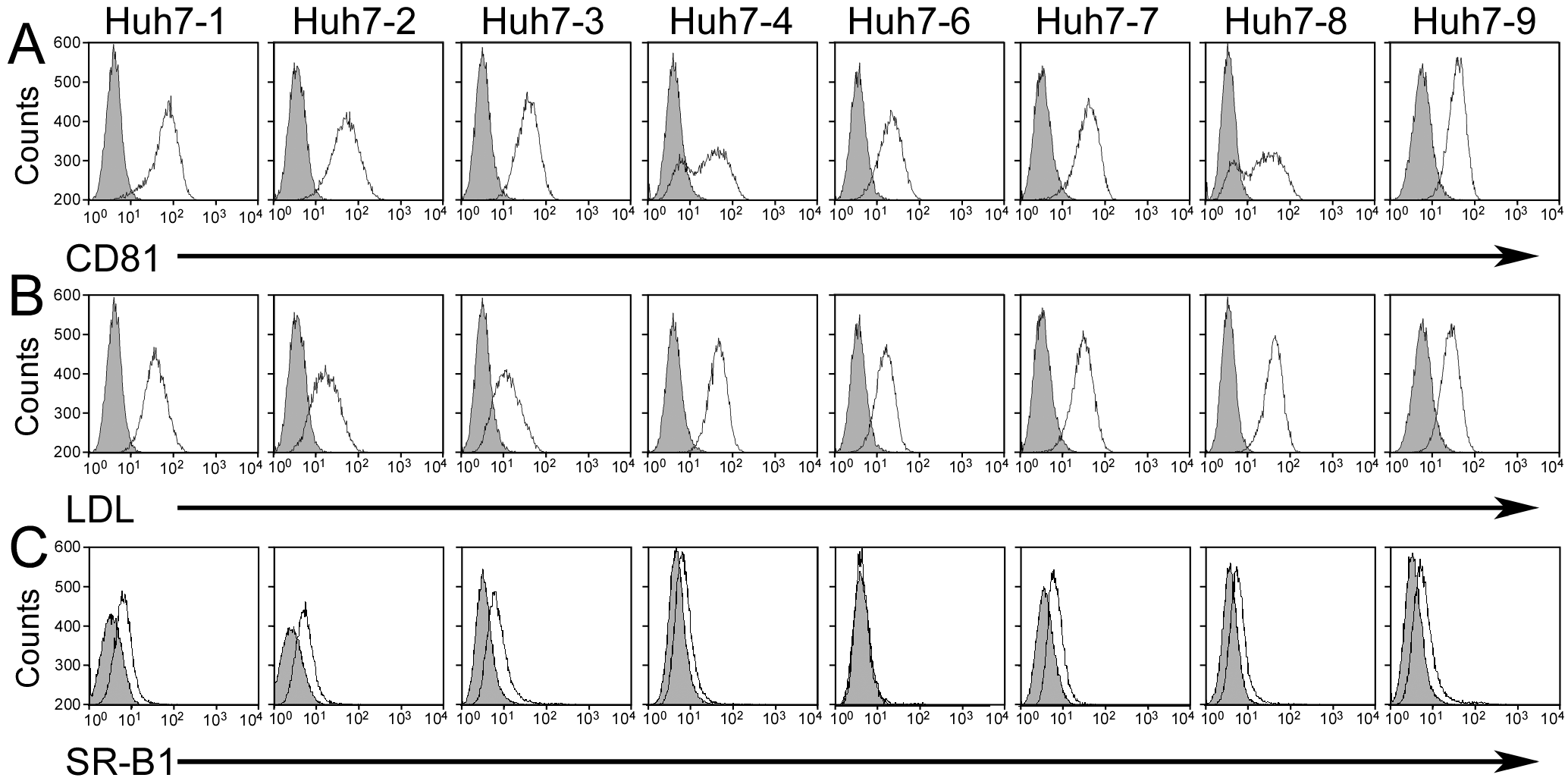

Supplement: Figure S3 — Flow cytometric analysis of HCV putative receptor surface expression on Huh7 cell lines. Each Huh7 cell line was stained with (A) mouse anti-CD81, (B) mouse anti-LDL-R or (C) rabbit anti-SR-B1 monoclonal antibodies and respective anti-mouse or anti-rabbit secondary antibodies conjugated with PE. Shaded regions represent cells stained with a monoclonal mouse or rabbit IgG control primary antibody and respective PE-conjugated secondary antibody. (2.14 MB TIF) [file pone.0006561.s005.tif]
